# Silencing of UBE2D1 inhibited cell migration in gastric cancer, decreasing ubiquitination of SMAD4

**DOI:** 10.1186/s13027-021-00402-2

**Published:** 2021-11-07

**Authors:** Honghu Xie, Yu He, Yugang Wu, Qicheng Lu

**Affiliations:** Department of Gastrointestinal Surgery, The First People’s Hospital of Changzhou, 185 Juqian Street, Changzhou, 213000 Jiangsu China

**Keywords:** UBE2D1, SMAD4, EMT, GC

## Abstract

**Background:**

Gastric cancer (GC) is the second leading cause of cancer-related deaths. Because it is hard to diagnose at early stage, the overall 5 years survival rate is lower than 25%. High migration is the main hallmark of malignant cells at advanced stage of GC. Thus, it is urgent to find biomarkers for early diagnosis and more effective therapy of GC.

**Methods:**

In this study, lentivirus-mediated silencing and overexpression lentiviruses targeting the ubiquitin-conjugating enzyme E2 D1 (UBE2D1), transwell, wound healing, and pulmonary metastasis mouse model were applied to analyze the function of UBE2D1 in vitro and in vivo. Real-time PCR and immunohistochemistry were used to elucidate the level of UBE2D1 in GC samples.

**Results:**

Silencing of UBE2D1 inhibited cell migration and the levels of epithelial-mesenchymal transition makers (MMP2 and MMP9) in AGS and MKN45 cells. Silencing of UBE2D1 inhibited cell metastasis in mouse model. On the contrary, UBE2D1 overexpression increased cell migration and the levels of MMP2 and MMP9 in MGC-803 cells. Further, silencing of UBE2D1 decreased the ubiquitination level of mothers against decapentaplegic homolog 4 (SMAD4), and the increase of cell migration induced by UBE2D1 overexpression could be reversed by SMAD4.

**Conclusion:**

Silencing of UBE2D1 inhibited cell migration through transforming growth factor β (TGF-β)/SMAD4 signaling pathway in GC.

**Supplementary Information:**

The online version contains supplementary material available at 10.1186/s13027-021-00402-2.

## Background

Gastric cancer (GC), a common malignant tumor of the digestive system, is the second leading cause of cancer-related deaths, with an overall 5 years survival rate lower than 25% [1]. The infectious agents including *Helicobacter pylori* and Epstein-Barr virus, and genetic factors including somatic mutations, mismatch repair gene deficiency, gene amplifications, and deletions are widely accept as the main causes of GC [2, 3]. As most of the patients are diagnosed at an advanced stage, those with GC receive limited benefit from radiotherapy, a standard treatment of GC [4]. Although many genes have been found to play a role in GC, the underlying mechanisms still need to be clarified. Therefore, it is necessary to explore biomarkers for early diagnosis and more effective therapy of GC.

Transforming growth factor β (TGF-β) signaling pathway is of great importance in regulating a large number of biological processes, including cell proliferation, apoptosis, differentiation, migration, and the initiation and progression of cancer [5]. Mothers against decapentaplegic homolog 4 (SMAD4), the central mediator of TGF-β signaling, plays a vital role in signal transferring from cell membrane to nucleus [6]. The canonical TGF-β/SMAD4 signaling pathway has been demonstrated to be a tumor suppresser in many cancer types including GC [6]. Recently, researchers revealed that the activation of TGF-β/SMAD4 signaling pathway promotes Epithelial-mesenchymal transition (EMT), which is considered as the transformation from epithelial cells to mesenchymal cells with the migratory and invasive abilities [7]. EMT contributes to a main part for cancer metastasis and transforming to mesenchymal cells has been found to enhance chemotherapy resistance and lead to a poor prognosis [8]. Therefore, it is helpful to explore biomarkers to inhibit EMT in cancer therapy.

The ubiquitin-conjugating enzyme E2 D1 (UBE2D1) is a member of UBE2D family which has been found to play a role in some important pathways of carcinogenesis [9, 10]. UBE2D1 has been demonstrated to participate in the degradation and ubiquitination of tumor suppressor protein p53 [10]. Further, UBE2D1 is involved in the deregulation of Wnt and Ras-MAPK signaling pathways influenced by Aurora kinase A in colorectal cancer [11]. It is reported that UBE2D1 together with cellular inhibitor of apoptosis protein 1 (c-IAP1) mediates the activation of NF-κB signaling pathway and receptor-interacting protein 1 (RIP1) ubiquitination stimulated by tumor necrosis factor α (TNFα) [12]. Researchers suggest that UBE2D1 may exhibit great importance in the regulation of cancer-related signaling pathways. Recently, UBE2D1 has been indicated to be a potential biomarker for the therapy of gastric cancer [13]. Thus, it is urgent to figure out the function of UBE2D1 in GC.

In the current study, we revealed that silencing of UBE2D1 suppressed cell migration in vitro and in vivo. In addition, TGF-β/SMAD4 signaling pathway was involved in the function of UBE2D1 in GC.

## Materials and methods cell lines and culture

Human GC cell lines, including AGS, BGC-823, MGC-803, MKN45, SGC-7901, and human normal gastric epithelial cell line GES-1 were purchased from iCell Bioscience Inc. (Shanghai, China). AGS, BGC-823, and MGC-803 were cultured in DMEM medium, and MKN45, SGC-7901, and GES-1 were cultured in RPMI-1640 medium. All the mediums were purchased from Gibco (Carlsbad, CA, USA) and 10% fetal bovine serum (Gibco) and 1% penicillin-streptomycin sulfate (Gibco) were added. 10 μM of MG132 (Selleckchem, Radnor, PA, USA), a proteasome inhibitor, or 100 μg/ml of cycloheximide (Selleckchem), a protein synthesis inhibitor were used to treat cells.

### Real-time PCR

TRIzol reagent (Thermo Fisher Scientific Inc, Grand Island, NY, USA) was used to extract cellular RNA and PrimerScript RT reagent Kit (Applied Biosystems, Foster City, CA, USA) was used to synthesize cDNA. SYBR Green kit was used to perform the PCR reactions in a 7500 Fast Real-time PCR System (Thermo Fisher Scientific). Relative gene expression levels were calculated using the 2^−ΔΔCT^ method and normalized to β-actin. The primer sequences are presented below.

$$\begin{array}{*{20}l} {{\rm{UBE2D1}}} \hfill & {\rm{Primer F}} \hfill & {{5}^{\prime}\;{\rm{AGCGCATATCAAGGTGGAG 3}}^{\prime}} \\& {\rm{Primer R}} & {{5}^{\prime}\;{\rm{AGAGCTGGTGACCATTGTG 3}}^{\prime}} \\ {{\rm{SMAD4}}} & {\rm{Primer F}} & {{5}^{\prime}\;{\rm{GCATTCCAGCCTCCCATTTCC 3}}^{\prime}} \\ & {\rm{Primer R}} & {{5}^{\prime}\;{\rm{GGCACCTGACCCAAACATCAC 3}}^{\prime}} \\ {\beta-{\rm{actin}}} & {\rm{Primer F}} & {{5}^{\prime}\;{\rm{GATGACCCAGATCATGTTTGAG 3}}^{\prime}}\\ & {\rm{Primer R}} & {{5}^{\prime}\;{\rm{TAATGTCACGCACGATTTCC 3}}^{\prime}}\\ \end{array}$$

### Western blot assay

The treated cells were lysed in RIPA buffer (Invitrogen, Carlsbad, CA) on ice. After 12,000 rpm centrifugation at 4 °C, a BCA kit (Beyotime, Shanghai, China) was used to measure protein concentration. Proteins (30 μg) from different samples were separated by 10% SDS-PAGE and blotted onto PVDF membranes (Millipore Corp., Bedford, MA, USA). After blocking in 5% non-fat milk, PVDF strips were incubated overnight with primary antibodies, followed by incubation with secondary antibodies. Primary antibodies were listed as following: UBE2D1 (1:800, Proteintech, Rosemont, IL, USA), SMAD4 (1:1000, Abcam, St. Louis, MO, USA), MMP2 (1:1000, Abcam), MMP9 (1:800, Abcam), and β-actin (1:2000, Abcam). At last, the membranes were scanned using a Bio-Rad imaging system (Hercules, CA, USA).

### Lentivirus construction

All these lentiviruses were constructed by Genechem company (shanghai, China), including three interfering (shUBE2D1-1: GCTGAAGAGGATTCAGAAA; shUBE2D1-2: GCGCATATCAAGGTGGAGT; shUBE2D1-3: CCAAAGATTGCTTTCACAA) and one overexpression lentiviruses targeting UBE2D1, and one SMAD4 overexpression lentivirus. Briefly, shRNA oligos targeting UBE2D1 were annealed and cloned into AgeI- and EcoRI-digested pLKO.1 (Addgene, Cambridge, MA, USA). The full-length of UBE2D1 and SMAD4 were separately cloned into pLVX-puro (Clontech, Palo Alto, CA, USA). The lentivirus was produced in 293 T cells along with packaging plasmids, psPAX2 and pMD2.G.

### Transwell assay

The transwell chambers (Corning Incorporated, Corning, NY, USA) were set into 24-well plates and incubated with culture medium overnight. Treated cells were harvested with FBS-free culture medium and seeded into the upper chamber at 2 × 10^4^ cells per well (about 200 μl). Next, the culture medium containing 10% FBS was added to the lower chamber and cultured for 24 h. After removing the non-migratory cells, the chambers were stained with 1% crystal violet (Sigma, St. Louis, MO, USA) for 15 min, followed by acquiring images (200 ×) using a microscope (Leica, Germany).

### Wound healing assay

A total of 1 × 10^5^ treated cells were planted into each well of a 6-well plate and cultured to 80% confluence. Then, a 200 µL pipette tip was used to create wounds in the middle of each well. After washing with PBS, the cells were incubated with culture medium containing 1% FBS. Images (200 ×) of wounds were acquired at 0 and 24 h. Each experiment was performed in triplicate.

### Pulmonary metastasis mouse model

A total number of 12 BALB/c nude mice (4 weeks old, male) were purchased from Shanghai SLAC Laboratory Animal Co.,Ltd (20170005030081, Shanghai, China). Mice were randomly divided into two groups: AGS-shNC and AGS-shUBE2D1. The cells transduced with shNC or shUBE2D1 lentivirus were harvested and suspended with PBS, then 1 × 10^6^ cells were subcutaneously injected into the tail vein of nude mice. After 4 weeks of feeding, all these mice were euthanized. The image of lung profile was acquired, and the metastatic nodules were counted. Then, the lung samples were fixed in 4% paraformaldehyde (Beyotime) for the hematoxylin–eosin staining. All the animal protocols were approved by the Institutional Animal Ethics Care and Use Committee of The First People’s Hospital of Changzhou. This study was performed in line with the principles of the Declaration of Helsinki.

### Gene set enrichment analysis (GSEA)

GSEA is used to analyze gene expression data using biological knowledge [14]. Lots of the studies were published using GSEA to analyze target gene expression [15–17]. In the current study, the Cancer Genome Atlas Stomach Adenocarcinoma (TCGA-STAD) datasets was analyzed by GSEA software (v2.0.13; Broad Institute, Cambridge, USA) following the detailed protocol on website (http://www.broad.mit.edu/gsea). GSEA was used to generate an ordered list of genes according to their correlation to UBE2D1 expression. The phenotype marker was UBE2D1-high versus UBE2D1-low.

### Hematoxylin-eosin (HE) staining

After fixing in 4% paraformaldehyde, the lung samples were paraffin-embedded, sliced (4 µm), baked, and stained with Hexion and E staining kit (Bio-Rad Laboratories) according to the manufacturer’s protocol. Images were acquired by light microscopy (Leica, Germany).

### Co-immunoprecipitation (Co-IP)

Treated cells were lysed as previously described [18]. Whole cellular lysates were obtained through RIPA lysis buffer and incubated with anti-UBE2D1 (Proteintech, 15475-1-AP) or anti-SMAD4 (Abcam, ab230815) at 4 °C overnight, followed by incubation with protein A/G PLUS-Agarose beads (Bio-Rad Laboratories) at 4 °C for 2 h. Then, the proteins which bound to beads were eluted and detected by western blot using antibodies against SMAD4 or UBE2D1.

### Immunohistochemistry (IHC)

Human gastric tissue microarray (Avilabio.com, Sanxi, China) was deparaffinized, rehydrated, antigen retrieved with 3% H_2_O_2_ in methanol, blocked, and incubated with anti-UBE2D1 antibody at 4 °C overnight. After washing twice with PBS-T for 10 min each, the slice was incubated with second antibody 1 h and DAB buffer for detection. Images were acquired by light microscopy (Leica, Germany).

### Statistical analysis

All data are reported as means ± standard deviation (SD). Student’s t-test and one-way ANOVA were used in data analysis. Survival curves were calculated using the Kaplan–Meier method and *P* < 0.05 was considered significant.

## Results

### UBE2D1 is elevated in GC tissues and correlated with poor prognosis

**Fig. 1:**
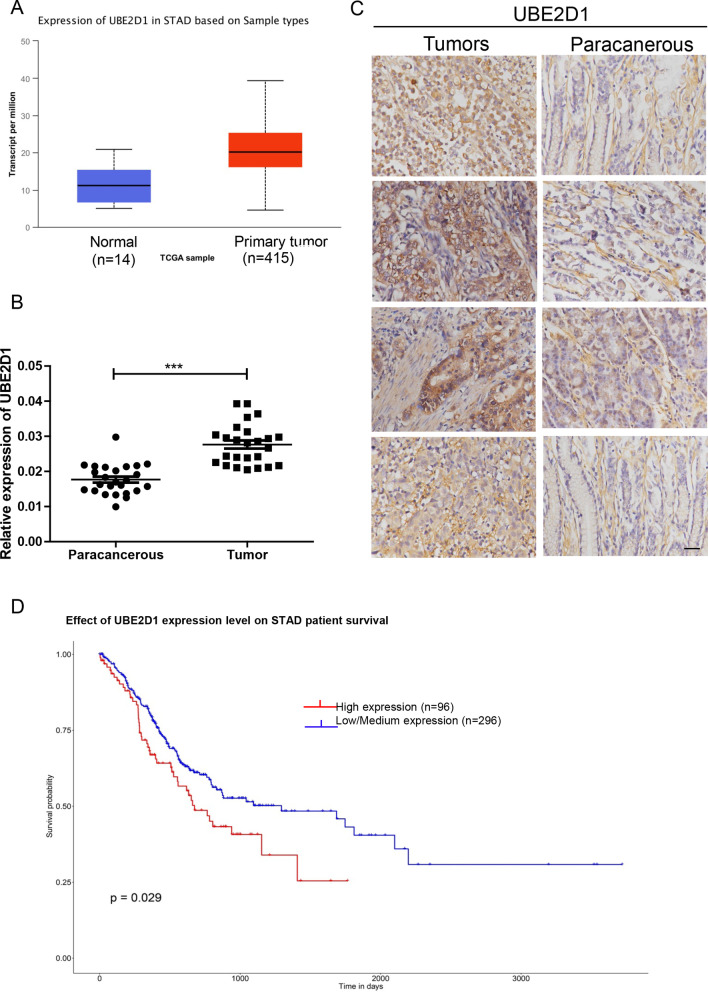
UBE2D1 was elevated in GC tissues. **A** The mRNA level of UBE2D1 was analyzed from TCGA-STAD data. **B** The mRNA level of UBE2D1 was detected in 25 pairs of tumors and paracancerous samples. ***, *P* < 0.001. **C** The protein level of UBE2D1 in tissue array (32 pairs) was determined by IHC staining. **D** Impact of UBE2D1 expression on OS in GC patients in TCGA

To determine the significance of UBE2D1 in GC, we analyzed the expression level of UBE2D1 in TCGA-STAD and found that UBE2D1 was upregulated in primary tumor (n = 415) compared to the normal tissue (n = 14, Fig. 1A). Further, UBE2D1 expression level was examined using real-time PCR in 25 pairs of tumors and paracancerous samples. Similarly, UBE2D1 level was higher in the tumor tissues than the paracancerous tissues (Fig. 1B). According to the results of IHC staining with GC tissue array (Fig. 1C), GC tumors (68.75%) expressed much higher protein level of UBE2D1 than the paracancerous tissues. In addition, the Kaplan–Meier survival curve was drew using the data from TCGA and demonstrated (Fig. 1D) that GC patients with high UBE2D1 mRNA expression exhibited a poor overall survival, indicating that UBE2D1 was dramatically associated with shortened GC patients’ overall survival (*P* = 0.029).

### Silencing of UBE2D1 suppresses cell migration

**Fig. 2:**
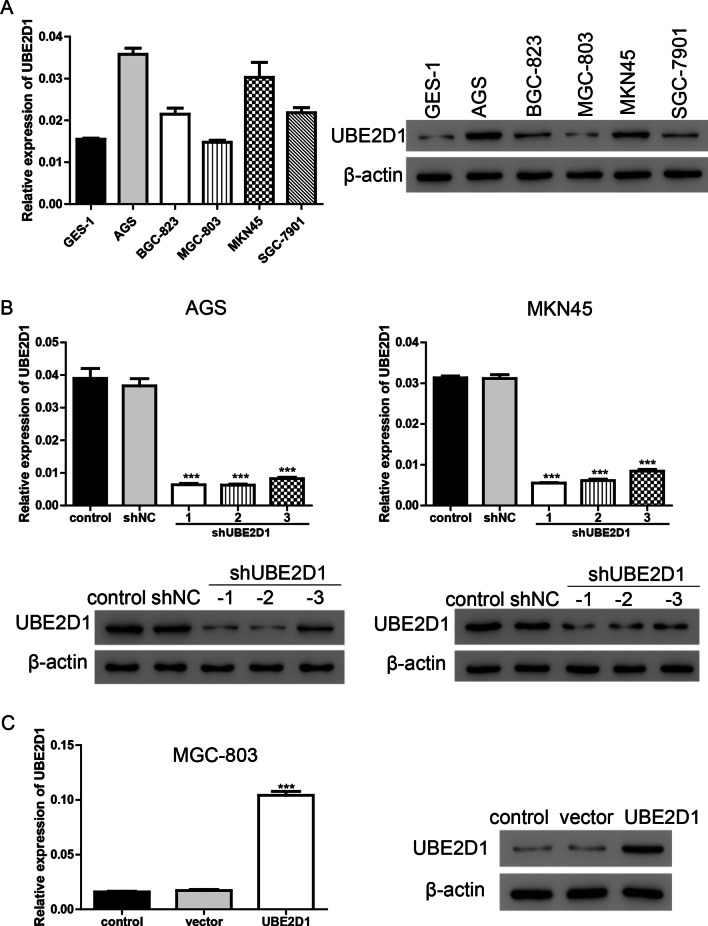
Expression level of UBE2D1 was measured by real-time PCR western blot assay. **A** UBE2D1 expression level in different cell lines, including GSE-1, AGS, BGC-823, MGC-803, MKN45, and SGC-7901. **B** Knock-down efficiency was evaluated after transduction with shUBE2D1 lentiviruses in AGS and MKN45 cells. ***, *P* < 0.001 versus shNC. **C** Overexpression efficiency was evaluated after transduction with UBE2D1 overexpression lentivirus in MGC-803 cells. ***, *P* < 0.001 versus vector

To explore the function of UBE2D1, we firstly measured its expression in human normal gastric epithelial cell line GES-1 and GC cell lines AGS, BGC-823, MGC-803, MKN45, and SGC-7901. As shown in Fig. 2A, UBE2D1 was upregulated in most GC cell lines expect MGC-803 in comparison with GES-1. Then, knock-down and overexpressing lentiviruses targeting UBE2D1 were applied to manipulate the expression level of UBE2D1. As expected, UBE2D1 was downregulated after transduction with shUBE2D1 lentiviruses in AGS and MKN45 cells which exhibited higher level of UBE2D1 (Fig. 2B). Meanwhile, UBE2D1 was upregulated after transduction with UBE2D1 overexpressing lentivirus in MGC-803 cells which exhibited lower level of UBE2D1 (Fig. 2C).

**Fig. 3:**
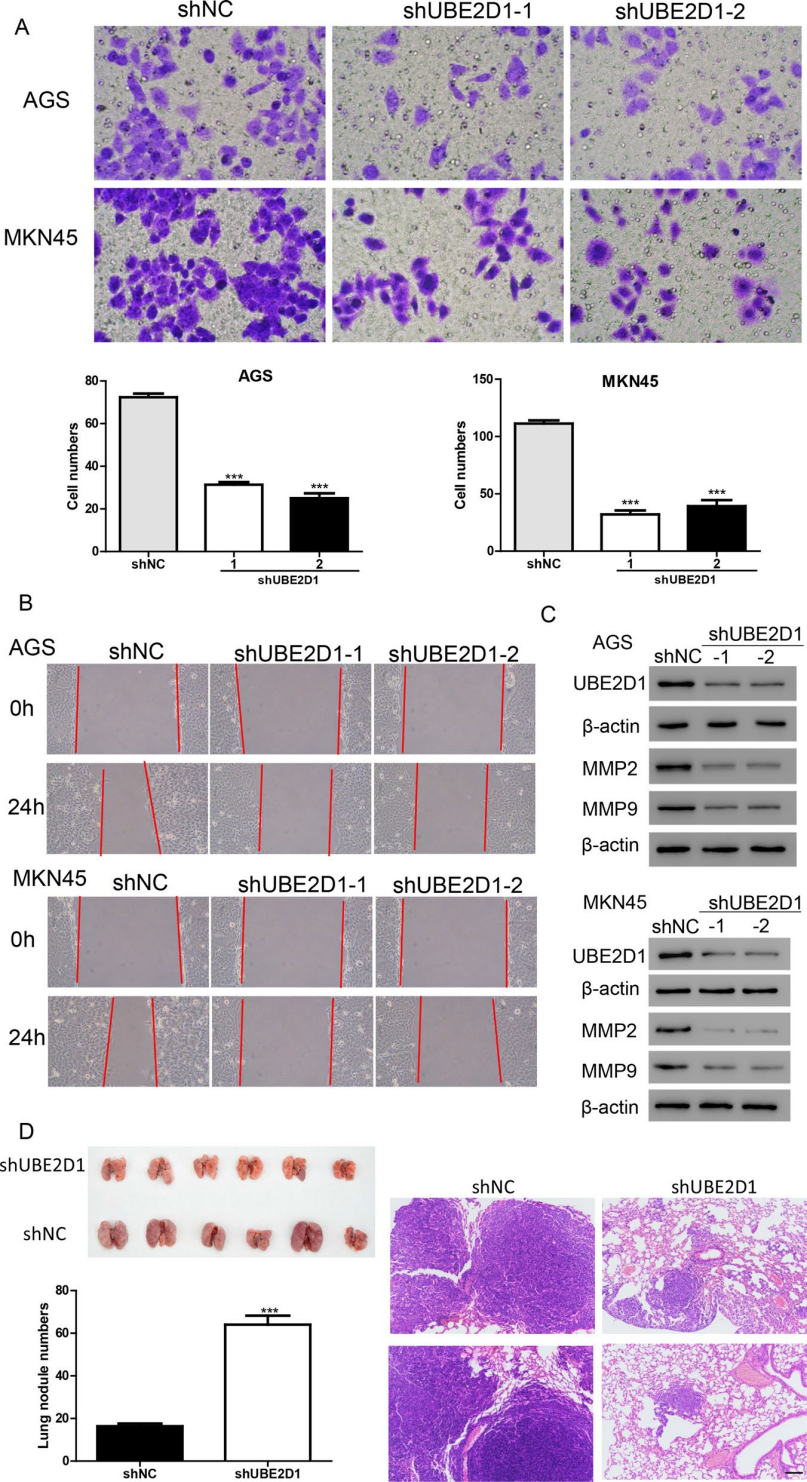
Silencing of UBE2D1 suppressed cell migration. **A**, **B** Transwell (**A**) and wound healing **B** assays were performed to analyze cell migration after transduction with shUBE2D1 lentiviruses in AGS and MKN45 cells. ***, *P* < 0.001 versus shNC. **C** A western blot assay was performed to measure the protein levels of UBE2D1, MMP2, and MMP9. **D** The lung metastatic nodules were much less in the shUBE2D1 mice than those in the shNC mice. ***, *P* < 0.001 versus shNC. Scale: 100 µm

Based on the result of transwell and wound healing assays (Fig. 3A, B), cell migration was remarkably inhibited after transduction with shUBE2D1 lentiviruses in AGS and MKN45 cells. Matrix metalloproteinases (MMPs) has been demonstrated to promote the migration cancer cells [19]. In this study, western blot assays were performed to detect the protein levels of MMP-2 and MMP-9 in the cells which were transduced with shUBE2D1 lentiviruses. Silencing of UBE2D1 significantly decreased the protein levels of MMP-2 and MMP-9 in AGS and MKN45 cells (Fig. 3C). In addition, UBE2D1 knockdown increased the level of E-cadherin but decreased the level of N-cadherin (Additional file 1: Fig. S1). Further, pulmonary metastasis mouse model was implemented. We found that the lung metastatic nodules were much less in the shUBE2D1 mice than those in the shNC mice. HE staining subsequently confirmed that silencing of UBE2D1 inhibited lung metastasis (Fig. 3D). Taken together, these results indicated that knock-down of UBE2D1 inhibited migration in vitro and in vivo.

### Overexpression of UBE2D1 promotes cell migration in vitro

**Fig. 4:**
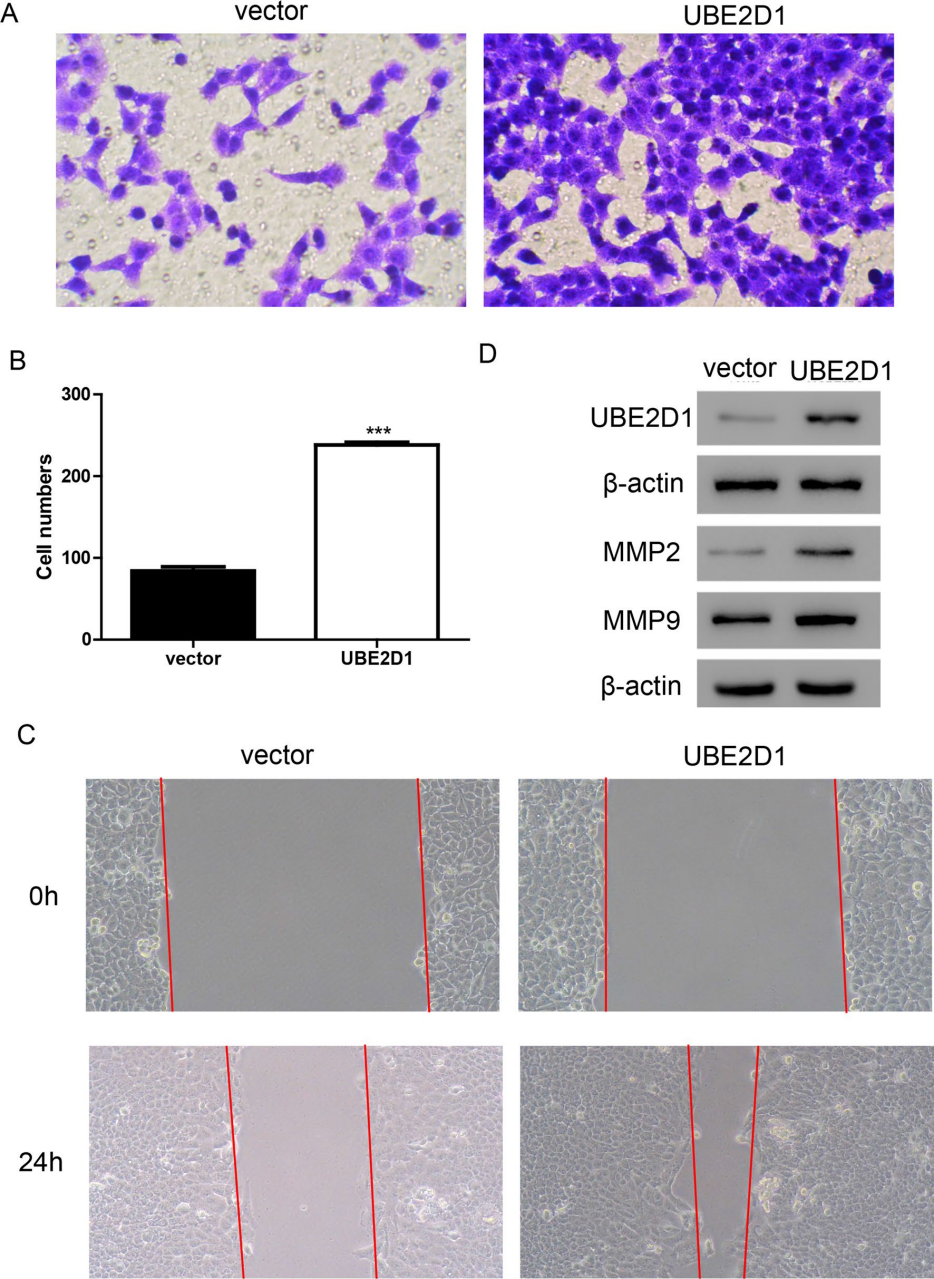
Overexpression of UBE2D1 promoted cell migration in vitro. **A**–**C** Transwell (**A** and **B**) and wound healing (**C**) assays were performed to analyze cell migration after transduction with UBE2D1 overexpression lentivirus in MGC-803 cells. ***, *P* < 0.001 versus vector. **D** A western blot assay was performed to measure the protein levels of UBE2D1, MMP2, and MMP9

To further confirm the function of UBE2D1, overexpressing lentivirus targeting UBE2D1 was used to treat MGC-803 cells. As shown in Fig. 4A, B, the migrating cells were much more in UBE2D1 overexpression group than those in the vector group. Wound healing assay demonstrated that UBE2D1 markedly increased cell migration (Fig. 4C). In addition, UBE2D1 significantly increased the protein levels of MMP2 and MMP9 in MGC-803 cells (Fig. 4D).

### Silencing of UBE2D1 decreases the ubiquitination level of SMAD4

**Fig. 5:**
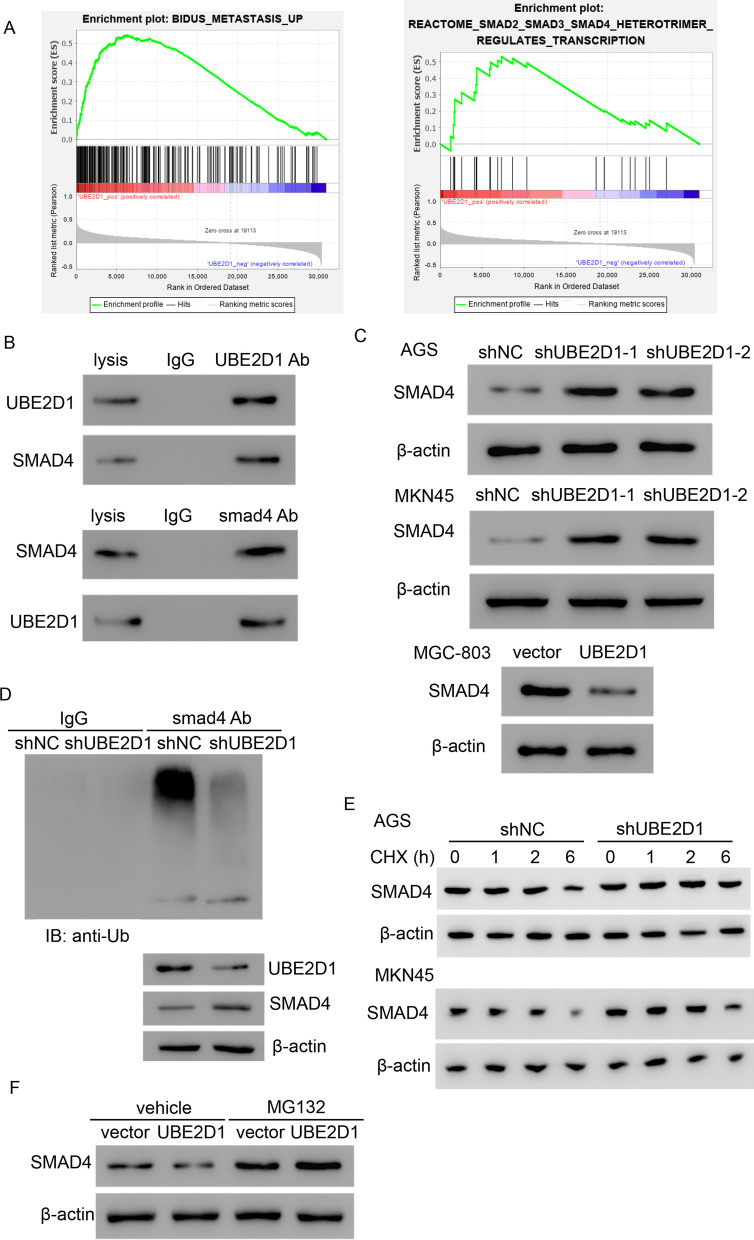
Silencing of UBE2D1 decreased the ubiquitination level of SMAD4. **A** UBE2D1 was identified to associate with metastasis and SMAD signaling pathways using GSEA. **B** Co-Ip assay was performed to detect the interaction between UBE2D1 and SMAD4. **C** The protein level of SMAD4 was measured after transduction with shUBE2D1 or overexpression lentivirus. **D** The ubiquitination level of SMAD4 was measured after transduction with shUBE2D1 lentivirus. **E** SMAD4 was detected after transduction with shUBE2D1 lentivirus upon treatment with 100 μg/ml CHX. **F** SMAD4 was detected after transduction with UBE2D1 overexpression lentivirus and/or 10 μM of MG132

GSEA, a method of interpreting gene expression data using biological knowledge [14], has been used to analyze gene expression in lots of studies [15–17]. Herein, we used GSEA to explore the UBE2D1-associated pathways. As shown in Fig. 5A, high expression of UBE2D1 was significantly associated with metastasis. After further analysis, the SMAD pathway was found to be associated with UBE2D1.

SMAD4 is the central mediator of TGF-β signaling, thus we detected whether there was a direct interaction between UBE2D1 and SMAD4 using Co-Ip assay. As we expected, UBE2D1 could interacted with SMAD4 directly and vice versa (Fig. 5B). Next, knock-down of UBE2D1 significantly increased the protein level of SMAD4 in both AGS and MKN45 cells. While overexpression of UBE2D1 decreased SMAD4 in MGC-803 cells (Fig. 5C). UBE2D1 is a ubiquitin-conjugating enzyme and is involved in many signaling pathways. Therefore, we measured the ubiquitination level of SMAD4 after transduction with shUBE2D1 lentivirus and found that silencing of UBE2D1 markedly inhibited the ubiquitination level of SMAD4 (Fig. 5D). To eliminate the effect of protein synthesis, 100 μg/ml cycloheximide was applied to cells. Similarly, SMAD4 level was upregulated by UBE2D1 knockdown upon cycloheximide treatment (Fig. 5E). In addition, 10 μM of MG132 was applied to MGC-803 cells. As shown in Fig. 5F, the decrease of SMAD4 induced by UBE2D1 could be blocked by MG132. In summary, these results indicated that UBE2D1 regulated the SMAD4 pathway by increasing the ubiquitination level of SMAD4.

### The increase of cell migration induced by UBE2D1 is alleviated by SMAD4

**Fig. 6:**
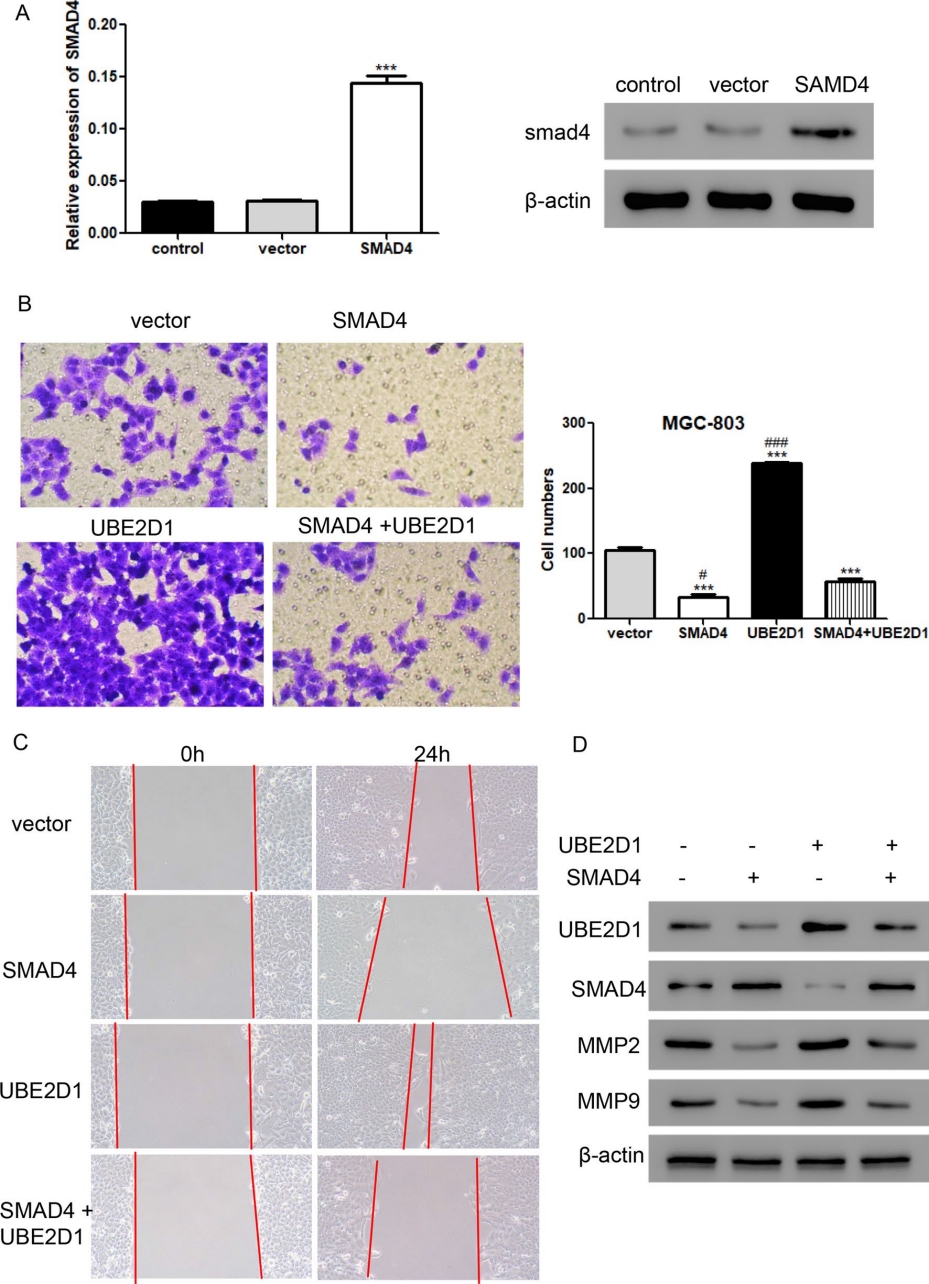
The increase of cell migration induced by UBE2D1 was reversed by SMAD4. **A** The mRNA and protein levels of SMAD4 were detected after transduction with SMAD4 overexpression lentivirus using real-time PCR and western blot. ***, *P* < 0.001 versus vector. **B**–**C** Transwell (**B**) and wound healing **C** assays were performed to analyze cell migration after transduction with SMAD4 and/or UBE2D1 overexpression lentiviruses in MGC-803 cells. ***, *P* < 0.001 versus vector; #, *P* < 0.05, ###, *P* < 0.001 versus SAMD4 + UBE2D1. **D** A western blot assay was performed to measure the protein levels of UBE2D1, SMAD4, MMP2, and MMP9

To further evaluate the function of UBE2D1 and SMAD4, SMAD4 overexpressing lentivirus was constructed to treat MGC-803 cells. As shown in Fig. 6A, both mRNA and protein levels of SMAD4 were significantly upregulated after transduction with SMAD4 overexpressing lentivirus. Transwell and wound healing assays (Fig. 6B, C) showed that cell migration was remarkably inhibited after transduction with SMAD4 overexpressing lentivirus. However, the increase of cell migration induced by UBE2D1 was alleviated by SMAD4 in MGC-803 cells. Further, we found that SMAD4 decreased the protein levels of MMP-2 and MMP-9, and co-treatment with SMAD4 and UBE2D1 overexpressing lentiviruses exhibited higher levels of MMP-2 and MMP-9 than the treatment with only SMAD4 overexpressing lentivirus but still lower than the treatment with only UBE2D1 overexpressing lentivirus (Fig. 6D). These data suggested that UBE2D1 regulated cell migration through TGF-β/SMAD4 signaling pathway.

## Discussion

GC, the second leading cause of cancer-related deaths, has a low overall 5 years survival rate and a high recurrence rate [20]. Due to the limitation of test makers and people’s cognition, most of the patients are diagnosed at an advanced stage. Unfortunately, the therapeutic effect for advanced GC with metastasis is far below expected [21]. In this study, we demonstrated that UBE2D1 was highly elevated in GC samples and high expression level of UBE2D1 associated with poor OS (Fig. 1). Silencing UBE2D1 inhibited cell migration in vitro and in vivo. This was the first time to report the function of UBE2D1 in GC cell migration. A high growth speed is the most obvious character of cancer cells, and migration contributes to the tumor malignance. Exploring novel makers to limit tumor cell migration is of great importance for cancer treatment.

Previous studies reported that upregulated UBE2D1 is found in several cancers including non-small-cell lung cancer [22], osteosarcoma [23], and hepatocellular carcinoma [24]. UBE2D1 might be a potential target for gene therapy for human gastric cancer [13]. In this study, real-time PCR of 25 pairs of GC and related paracancerous samples and tissue array results revealed that UBE2D1 was elevated in GC samples. Based on the data from TCGA and the analysis of Kaplan–Meier survival curves, high level of UBE2D1 was observed in GC samples and associated with poor survival outcomes. UBE2D1 belongs to UBE2D family which is demonstrated to be a critical mediator in the ubiquitination and degradation of p53 [9]. Ubiquitination plays crucial roles for physiological processes and regulates both tumor-promoting and tumor-suppressing pathways [25]. UBE2D1 has been found to trigger the ubiquitination of p53 by interacting with MDM2 as an E2s in vitro [26]. Recently, UBE2D1 has been revealed to participate in the ubiquitination of HSP90AB1, which mediates to the transportation and stability of p53 [24]. In the current study, UBE2D1 was found to regulate TGF-β/SMAD4 signaling pathway through ubiquitinating SMAD4.

The TGF-β/SMAD4 signaling pathway has a crosstalk with many other pathways and is famous in a plenty area, including cancer, EMT, microRNA, DNA damage response and DNA damage repair [6]. In cancer cells, EMT contributes the acquisition of migratory and invasive abilities, leading to the promotion of tumor development [7]. MMP-2 and MMP-9, which contribute to cell migration and invasion, have been found upregulated during EMT in cancer cells [27]. Here, the results showed that knock-down of UBE2D1 inhibited cell migration and also decreased the levels of MMP2 and MMP9 in GC cells. More importantly, UBE2D1 silencing decreased the ubiquitination level of SMAD4. Consistently, low level of SMAD4 has been found to associate with high MMP-9, resulting colorectal cancer malignance [28].

In conclusion, silencing of UBE2D1 decreased the ubiquitination and degradation of SMAD4, resulting the inhibition of cell migration in GC.

## Supplementary Information

**Additional file 1.** The protein levels of E-cadherin and N-cadherin were measure after transduction of shUBE2D1 lentiviruses in AGS and MKN45 cells.

## Data Availability

The data are available from the corresponding author upon reasonable request.

## Abbreviations

Co-IP: Co-immunoprecipitation
EMT: Epithelial-mesenchymal transition
GC: Gastric cancer
GSEA: Gene set enrichment analysis
HE: Hematoxylin-eosin
IHC: Immunohistochemistry
SMAD4: Mothers against decapentaplegic homolog 4
UBE2D1: Ubiquitin-conjugating enzyme E2 D1
