# Supplementary material for: Silencing of UBE2D1 inhibited cell migration in gastric cancer, decreasing ubiquitination of SMAD4
Source: Infect Agent Cancer. 2021 Nov 7;16:63. doi: 10.1186/s13027-021-00402-2 (PMC8574036; doi:10.1186/s13027-021-00402-2)
Supplement: Supplementary file 1 — Additional file 1. The protein levels of E-cadherin and N-cadherin were measure after transduction of shUBE2D1 lentiviruses in AGS and MKN45 cells. [file 13027_2021_402_MOESM1_ESM.docx]

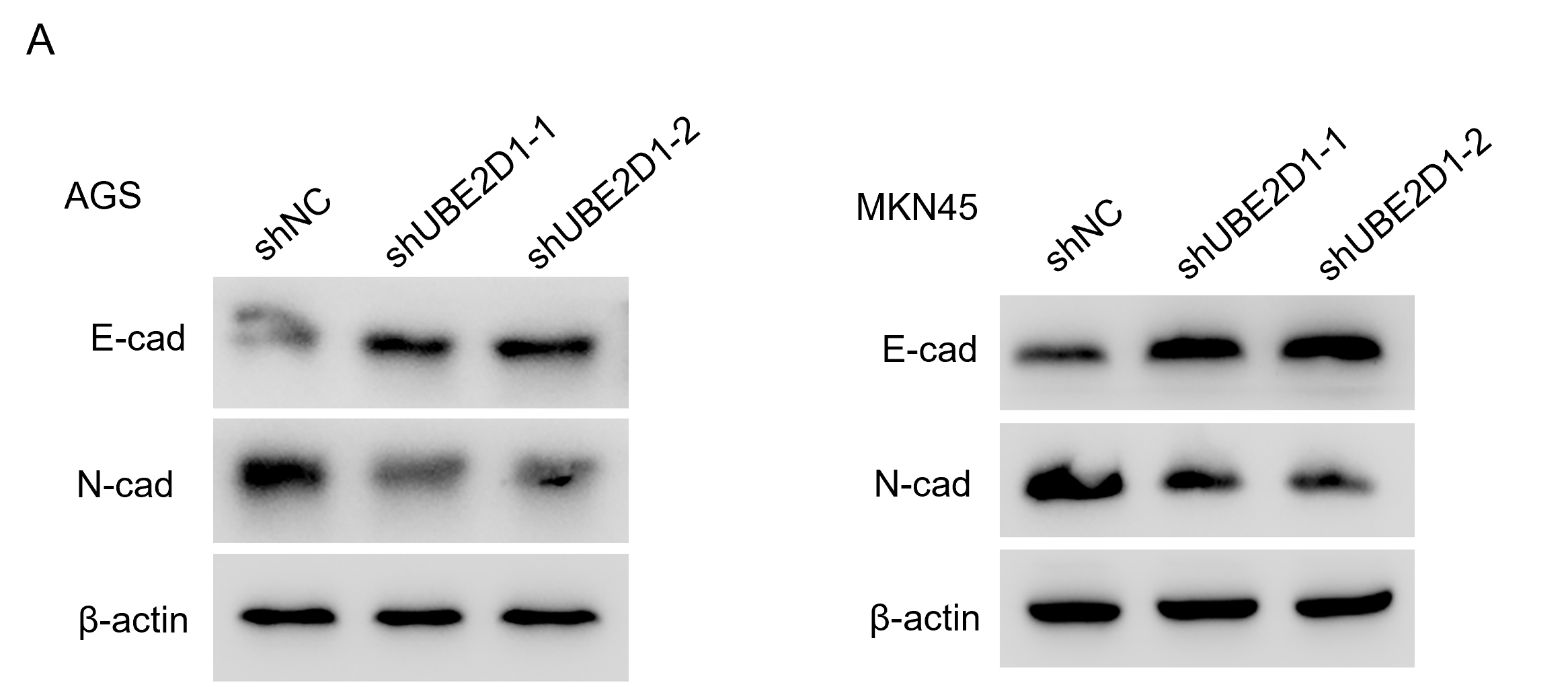


Supplemental figure. The protein levels of E-cadherin and N-cadherin were measure after transduction of shUBE2D1 lentiviruses in AGS and MKN45 cells.
